# How education, training and development support the wellness of employees with disabilities

**DOI:** 10.4102/ajod.v11i0.882

**Published:** 2022-04-29

**Authors:** Zelna van Niekerk, Mbulaheni O. Maguvhe, Meahabo D. Magano

**Affiliations:** 1 Department of Human Resource Management, College of Economic and Management Sciences, University of South Africa, Pretoria, South Africa; 2 Department of Inclusive Education, College of Education, University of South Africa, Pretoria, South Africa; 3 Department of Educational Psychology, College of Education, University of South Africa, Pretoria, South Africa

**Keywords:** development, disability, education, employees with disabilities, employers, equality, training, wellness

## Abstract

**Background:**

Existing wellness theories do not consider the unique needs of persons with disabilities. The lack of recognition of these needs in traditional wellness theories encouraged the researchers to develop a wellness framework for employees with disabilities (EWDs) to influence their wellness positively.

**Objective:**

The aim of the study was to identify the wellness experiences of EWDs and explore how education, training and development can contribute towards the employees’ wellness.

**Method:**

The qualitative study entailed semi-structured interviews with EWDs identified through snowball sampling. The study used the six-dimensional model of wellness that Bill Hettler developed in 1976 as a departure point to a holistic approach referring to social, intellectual, spiritual, physical, emotional and occupational wellness. The data collected was analysed through content analysis.

**Results:**

The study found that EWDs experience various workplace challenges as limited or no changes have been made to accommodate their specific needs. This then has a negative influence on their wellness. Their wellness diminishes as they attempt to cope with circumstances rather than request assistance. They recognised development needs in all the wellness dimensions explored. Employers and other stakeholders, including customers, colleagues and the communities they serve, need development and capacity building on disability matters to ensure equal opportunities for EWDs.

**Conclusion:**

The study resulted in a Wellness framework for EWDs identifying the education, training and development needs that will contribute to their wellness.

## Introduction

### Social value

In 2016, the International Labour Organization (ILO) reported that persons with disabilities (PWDs) represented half a billion of the world population (ILO 2016). This while the African Union (AU) in 2020 reported that nearly 1 in 10 Africans lives with one or another disability (African Union 2020), and in South Africa in 2019, 6.6% of the population older than 5 years were considered to have a disability (StatsSA 2019). The World Health Organization (WHO) (2016) also stressed that PWDs still experience unequal treatment in many instances including health care services. For example, ‘women with disabilities receive less screening for breast and cervical cancer than women without disabilities’, and ‘Adolescents and adults with disabilities are more likely to be excluded from sex education programmes’ (WHO 2016:1); this on the continent with the highest HIV infection rate.

This research study aimed to consider the current wellness levels experienced by employees with disabilities (EWDs) and how Education, Training and Development (ETD) can contribute to it. Therefore, the social value of this study lies in its potential to improve the wellness of EWDs in all the wellness dimensions identified by Hettler (National Wellness Institute 2014), which includes their social, intellectual, spiritual, physical, emotional and occupational wellness. An improvement of the holistic wellness of EWDs will result in an improvement in their general quality of life and their relationships (Marx 2015). Wellness in the workplace encompasses more than just arranging wellness initiatives. It also entails supporting employees and EWDS in building and maintaining relationships in the workplace and outside (Marx 2015; Walsh 2016).

Since the first democratic election in 1994, various pieces of legislation, White Papers and guidelines on disability were developed; however, the progress with the implementation and towards reaching the set targets have been disappointing. Considering this, it is understandable that the Commission for Employment Equity (CEE) indicated the following in its 2018–2019 Annual Report:

[*I*]t is noticeable from the trend analysis on the representation of Persons with Disabilities over the past three years that little progress is being made in increasing the representation of Persons with Disabilities in the workforce across all occupational levels. (CEE 2019:60)

**FIGURE 1:**
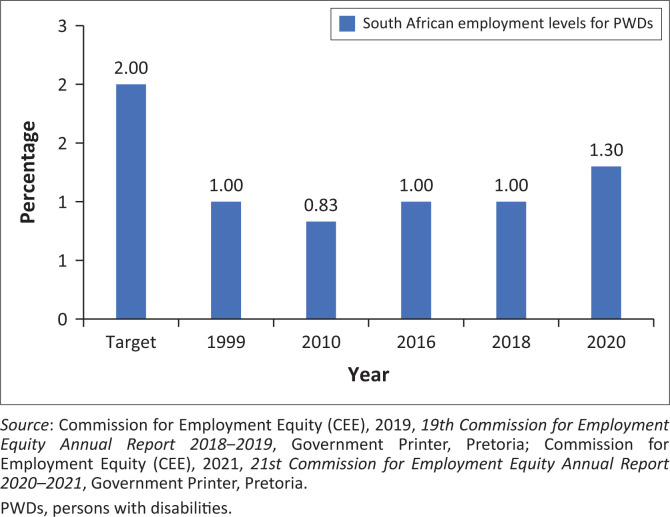
Employment levels for persons with disabilities (synthesised from employment equity reports released annually by the Commission for Employment Equity).

Figure 1 will give an overview of the employment levels of PWDs in South Africa. A target of 2% for the employment of PWDs was set in the White paper on Affirmative Action 1998, but by 2020, the national level of employment of PWDs was only 1.3% (CEE 2021):

With this lack of progress in mind, the scientific value of the study will now be discussed.

### Scientific value

According to the WHO (2016), the fact that PWDs still lack life and work skills is common all over the world. This lack of skills in turn has a negative impact on their wellness experiences, which are further relegated by the fact that their families, society and employers need development on how best to interact with PWDs. This indicates the need for a framework to help address the negative impact that limited skills have on the wellness of EWDs.

This inequality and discrimination negatively influence their wellness experiences, which is further relegated by the fact that their families, society, and employers do not know how to interact with them. (UN 2019; Van Niekerk & Van der Merwe 2013). To understand the variables in the study as a background to interpreting the personal experiences of the sample, various literary sources were studied during the literature review phase. It was important to acknowledge that PWDs are still excluded from society and the labour market (UN 2015; WHO 2016). The study considered various existing theories and models of both disability and wellness in the existing body of knowledge, but the wellness models do not acknowledge the unique needs of EWDs (Goss 2011; Van Niekerk & Van der Merwe 2013). The different disability and wellness models will now be discussed shortly as part of the theoretical framework for the study.

### Conceptual framework

To ensure a common understanding of the key concepts that formed part of the study in question, it will now be introduced before the relationships of these concepts with each other and the theory available will be investigated in more detail in the theoretical framework.

#### Disability

In the *Employment Services Act 4 of 2014*, PWDs are defined as

[*P*]ersons who have long-term physical, mental, intellectual, or sensory impairment, which, in interaction with various barriers, may hinder their full and effective participation in society on an equal basis with others. (Richardson 2017; Republic of South Africa (RSA), Department of Labour 2014a:4).

This definition is the same as the definition of the UN per the Convention on the Rights of Persons with Disabilities (CRPD) (UN 2006:4), which was upheld in the United Nations Disability Inclusion Strategy (UNDIS) (UN 2019).

In understanding disability in the workplace, it is also important to understand the types and causes of disability. Persons with disabilities include persons with physical, intellectual, mental or sensory disabilities (Services SETA 2006), some of which can be caused by environmental issues such as lead in drinking water (United States of America (USA) Environmental Protection Agency (EPA) 2015). Crime and the abuse of women and children are common in South Africa and Africa, which, with poverty and illiteracy, are considered prevalent societal factors that can result in disability. The lack of medical care and access to educational programmes further increases the occurrence of disability in African countries (Parrotte 2015; WHO 2015).

Lifestyle choices can also result in disability as high levels of obesity, alcohol abuse and other non-communicable diseases caused by lifestyle choices contribute to illness and disability (RSA, Department of Social Development (DSD), Department of Women, Children and Persons with Disabilities and United Nations International Children’s Emergency Fund – UNICEF 2012; Tomer 2016). Not only does obesity cause medical conditions like heart disease, but physically it also results in musculoskeletal strain, whilst alcohol abuse during pregnancy results in infants with disabilities because of foetal alcohol syndrome (Van Niekerk 2018; WHO 2016). Disabilities can also be hereditary or caused by accidents – in the USA work accidents are the major cause of disability, whilst in South Africa (WHO 2016) road accidents lead to high levels of permanent disability.

#### Wellness

According to the WHO’s constitution, wellness is ‘physical, mental, and social well-being, not merely the absence of disease’ (WHO 2015:2), whilst the National Wellness Institute refers to wellness as achieving self-actualisation and a full life (Hettler 1976; National Wellness Institute 2014). According to Dillette, Douglas and Andrzejewski (2021), Dunn, in 1959, asserted that holistic wellness refers to four dimensions which include body, mind, spirit and environment. As the researcher in this study focused on the wellness of EWDs, it was also important to consider how their work, the workplace and their growth influence their holistic wellness. According to Hettler and the National Wellness Institute, the effort by a person to achieve whole-person wellness is in line with Maslow’s theory on motivation which considers self-actualisation as the optimal level of meeting your needs (Maslow 1943; National Wellness Institute 2014).

### Theoretical framework

The following discussion will provide more information on these concepts and its relationship theory available on these key concepts and contextualise these concepts in relation to the research study.

#### Disability Model

In this study, the researcher used the bio-psycho-social (BPS) model of disability as the lens for viewing PWDs and EWDs. This approach allowed the researcher to consider all aspects of disability, from the person and the type of disability to the impact of society and the environment on them and their experiences (Krahn et al. 2006). Waddell and Aylward (2010) acknowledged that although disability is clearly linked to health, it also has social and psychological components that also play major roles in the wellness of PWDs (Shakespeare, Watson & Alghaib 2017; Waddell & Aylward 2010). Therefore, as a holistic model, it considers the impact of physical (biological or environmental) and emotional (psycho) factors, as well as the impact of society on PWDs (Waddell & Aylward 2010; Shakespeare et al. 2017). In summary, the BPS is accurately described by Hartley:

[*S*]ickness and disability are best overcome by an appropriate combination of healthcare, rehabilitation, personal effort and social or work adjustments. There is a coherent theory behind this assessment. (Hartley 2012)

#### Wellness Model

The researcher found Hettler’s (1976) six-dimensional model of wellness most suitable as an all-encompassing wellness model. In 1976, Hettler designed his six-dimensional model, which considers the ‘whole’ person and how these dimensions contribute to their work and home life. Although various wellness models exist, none of them refer to the specific needs of PWDs (Goss 2011). By using this model as the basis of the study, the researcher considered the holistic wellness of participants.

This model considers the physical, emotional, social, occupational, spiritual and intellectual dimensions of wellness and, in this specific study, its contribution to the holistic wellness of the employee with a disability. Physical wellness covers the body of a human being and its optimum functionality without any hindrance. Furthermore, one can use limbs and perform any duty to one’s satisfaction (Hettler 1976). For EWDs, the physical wellness has some limitations. Emotional wellness demonstrates the way an individual attains adequate fulfilment emotionally. One’s effective wellbeing is developed in such a way that one can handle any situation and accept any condition in a mature way. Social wellness demonstrates the ability to interact with other people in a healthy and acceptable way without any anti-social behaviour. Occupational wellness is attained when one’s optimum development in any career is self-actualised. In addition, one can demonstrate competency, creativity and resilience in one’s career. Spiritual wellness covers the belief system of an individual irrespective of one’s affiliation. Spiritual wellness includes the ability to exist with other people with different belief systems, and one can accommodate them because of one’s maturity. The sixth wellness dimension is the intellectual wellness that recognises creative abilities in applying knowledge and skills either in academic settings, professional development and community life (Hettler 1976).

A practical example of the impact of disability on the six wellness dimensions can be seen in the following scenario. According to WHO (2016), PWDs obtain limited education and job-related training because of prejudice and discrimination. This impacts on the intellectual development and wellness of PWDs as well as their occupational wellness. Such a situation also limits their career opportunities that directly impact on their income. This also has a negative impact on the emotional wellness of PWDs as WHO (2016) found that financial and occupational stress result in depression. Depression affects the social and spiritual wellness of people including PWDs and subsequently their physical wellness as depression is seen as the most common secondary disability. This clearly indicates how interrelated all wellness dimensions are as indicated by Hettler (1976).

This allowed the researcher to address the aims of the study, from considering the wellness status of EWDs to identifying the ETD support offered to and needed by EWDs and other role players, to develop an original wellness framework for EWDs.

#### Disability and wellness

Persons with disabilities were found to consider wellness as good physical and emotional health with manageable and limited physical pain (Oschwald & Powers n.d.). It also refers to the level of independence PWDs experience in their day-to-day living. It is the ability to do what you want even if in another way or with assistance and to make decisions for yourself (RSA, DSD 2015).

In 2014, the UN reported that more than 60% of African countries are still non-developed countries and subsequently have a lower quality of life and wellness (UN 2014). This came into play in this research study, as the WHO in 2011 and 2016 reported that PWDs have the highest levels of poverty in Africa as well as the highest levels of health issues (WHO 2016). As mentioned above, disability is prevalent in South Africa and Africa. Limited primary healthcare and poverty are not the only common causes of disability in Africa. Mining, which is common in Africa, especially in South Africa, increases acid mine-water poisoning. This is found where effluent or liquid waste (waste other than that from kitchens and toilets and produced by industries like the mining industry) flows into rivers and boreholes (Oruko et al. 2020). Furthermore, abuse and neglect of PWDs are common practices in Africa (African Union 2014; RSA & Presidency 2014; WHO 2016).

However, initiatives for wellness of PWDs are not limited to Africa. Internationally the UN launched the World Programme of Action Concerning Disabled Persons in 2015. This programme plans to address the plight of PWDs worldwide to ensure better living conditions, quality of life and opportunities. The UN also wants to prevent disabilities where possible or offer rehabilitation and reasonable accommodation where needed and thus ensure the well-being of PWDs (UN 2015). This is not only in line with wellness through ‘self-actualisation’ as mentioned above but also with the disability and wellness theories used as a lens for this study.

### Aims and objectives

The aim of this study was to explore the experiences of EWDs in terms of all wellness dimensions and to determine what ETD interventions organisations can offer EWDs and other stakeholders to improve the holistic wellness of EWDs.

## Research methods and design

### Study design

The interrelated research questions considered in this study are as follows: What kind of education training and development support is needed in organisations to improve the wellness of EWDs? How should this be contained in a wellness framework for EWDs?

### Research paradigm

As the research study concentrated on the experiences of PWDs and their wellness experiences in the workplace, it was situated in the interpretivist paradigm (Cohen, Manion & Morrison 2014). This paradigm also focuses on these experiences within set boundaries (Cohen et al. 2014). This correlates with the fact that this study applied a phenomenological research approach considering the wellness experiences of a specific group, namely PWDs, within a specific environment – the workplace – and supported by workplace training and development. However, it still allowed the researcher to study different interpretations of these experiences by interviewing different participants in different organisations and different industries (Cohen et al. 2014; Lincoln & Guba 1985).

All the above linked directly to the qualitative research methodology used in this study, focusing on the personal experiences of each participant in their own environment (Creswell 2014; Pugsley 2010). Qualitative research in this case improved the understanding of a social phenomenon – the wellness of EWDs; however, the findings cannot be generalised (Creswell 2014).

### Study population and sampling strategy

The study population consisted of PWDs employed in seven companies. As the population of EWDs is small and disability status is confidential, the primary researcher communicated with gatekeepers in each organisation who forwarded an introduction letter to EWDs. Snowball referencing as a non-intrusive sampling method (Baltar & Brunet 2012) was suitable to the study as it placed no pressure on EWDs to participate in the study.

### Data collection

The data were collected by administering semi-structured interviews to 12 employees with different physical and sensory disabilities pre-coronavirus disease 2019 (COVID-19), as it allowed the researcher to obtain responses to standard questions and to gather rich, unanticipated data from responses to the open questions. Interviews were considered suitable for qualitative, phenomenological studies as they provide in-depth data on how a participant experiences certain circumstance. It also allows participants the opportunity to have questions repeated or clarified, whilst interviewers can ask follow-up questions to clarify responses received from participants (Creswell 2014; Wiersema & Jurs 2009). In this study, the semi-structured interview schedule with 15 open-ended questions was based on the six dimensions of Hettler’s model of wellness (1976) but focused specifically on the experiences of the EWDs and on how ETD can improve these experiences.

### Data analysis

Data analysis is not a simplistic evaluation of the data collected; in this qualitative study, it was an exhaustive process of content analyses creating a better understanding of the phenomenon studied (Cohen et al. 2014; Creswell 2014). The researcher also recorded the interviews, and afterwards, it was transcribed verbatim before the data was coded by the researcher and an independent co-coder using software to code known as Atlas Ti. During the content analysis process, meaning was deducted (Potter & Hepburn 2008) and collated, thereby forming the basis for all findings and conclusions of the study.

### Ethical considerations

The researcher took extra care in ensuring ethical research practices as PWDs are a vulnerable group (RSA, DOL 2014a; UN 2006). Firstly, the researcher obtained ethical clearance from the College of Education Research Ethics Committee (reference number: 2015/09/16/8423369/ 21/MC) and then from the employer-wide UNISA Research permission sub-committee of SRIHDC (reference number: 2016_RPSC_003). Permission was obtained from the relevant organisations to conduct the research and attain the details of the relevant gatekeeper. The researcher also obtained informed consent from each participant, and it was made clear that they could withdraw at any time with no penalty. The researcher offered anonymity to participants, and therefore the researcher undertook to use pseudonyms, for instance, Participant A and so forth during the transcription, coding and the reporting of the findings of the study in any subsequent publications.

The data furthermore informed the development of the wellness framework for EWDs, as well as the subsequent implementation model. During the data analyses and this development process, existing academic sources were also considered in supporting or negating the findings (Creswell 2014; Potter & Hepburn 2008).

## Key findings and discussion

The themes identified are reported and discussed in more detail. Verbatim quotes from the transcribed interviews are included using pseudonyms. The findings discussed are based on the codes and themes identified by the researcher and the co-coder and the correlations found unless otherwise indicated.

### Theme 1: To improve the wellness of employees with disabilities, employers need to offer disability-specific education, training and developmental support

The participants identified three different categories of developmental needs. These were the developmental needs of the individual (the EWD), followed by those of the organisation and, finally, the needs of the wider society.

#### Category 1: Personalised education, training and development is required for employees with disabilities

**Education, training and development:** Most of the participants indicated their need for formal education.

Participant A stated the following:

‘Like now, I need to look, I need to grow, I need to see myself somewhere … if I can see myself having a degree … you need to see a growth in your life.’

Whilst Participant B felt that:

‘I just need that support …, the inspiration that I need, and motivation … for me to continue studying ….’

This is in line with Kamal et al. (2012) who stress that full participation of employees in the workplace is dependent on their development. A lack thereof will have a negative impact on their self-image resulting in feelings of inferiority and even stress and depression (Bam & Ronnie 2019; Kamal et al. 2012; Kwarbai & Akinpelu 2016).

Another personal developmental need identified by participants was the training to help them function optimally in the workplace. Although many participants stressed the need for induction training, the need encompassed more; it also referred to task-specific skills.

For Participant B, orientation on the new premises of his organisation was very important:

‘… when we get there, they should also organise some … orientation there, … to show us around, how that building operate[*s*] ….’

Participant D stressed that he had no formal training on his specific tasks:

‘I was always like taught, “Watch and learn” … information is like half floating around, … trial and error, always have to assume, “Is this the right thing to do? Is this right what I’m doing?”’

To ensure the ETD of EWDs, organisations will have to change their view regarding EWDs and what they believe these employees can do (Bam & Ronnie 2019). Often, they are employed only to meet employment equity targets with little future investment in their development as employees (RSA, DOL 2014a; Van Niekerk & Van der Merwe 2013).

### Career development

In times of economic downturn, as is currently being experienced in South Africa and globally, it is common for training and development to be minimised (Erasmus et al. 2019). Then the value of on-the-job training, such as formalised programmes involving job rotation, or mentoring and coaching, cannot be underestimated in such circumstances (Erasmus et al. 2019). Structured workplace career development programmes and plans (Erasmus et al. 2019) also have an important role to play in employee development. Career plans have an important role in all employee development and for EWDs, like all other employees, it gives them a ‘map’ of where they are going and what to do to grow (Al Zidjaly 2016; Erasmus et al. 2019).

Both Participants E and J mentioned how the lack of career management and progression demotivated them and had a negative impact on their emotional and occupational wellness:

Participant E:

‘I’m now nearly 6 years as [*position*] … It’s just too long, because I’m now at a plateau. You can’t be a [*position*] so long, because it is tough, and it’s emotionally very, very tough ….’

And Participant J:

‘I did apply for the director’s job … I didn’t get it, and, afterwards, I was told that it was a very good interview, but the fact that I couldn’t drive was one of the factors that … counted against me. I wasn’t very impressed ….’

### Disability and coping skills

In 2016, the WHO stated that ‘[d]epression is the leading cause of [mental] disability worldwide’ (WHO 2016:1) preventing people from functioning effectively. It is also the most common secondary disability (a secondary condition to other disabilities), as PWDs face various physical and emotional barriers as well as prejudice (Falvo 2014).

Both Participants G and L referred to circumstances in the workplace that negatively affected their emotional wellness:

Participant G:

‘No, I feel that the process [Incapacity process] I went through, I wouldn’t want somebody else to go through the same process. Emotionally, and there was a stage where, … I was battling to accept it. I actually went through depression.’

And Participant L:

‘It was far from home [place of transfer], so there was no one I know that side, and it became a problem for me to …, I am still … staying alone, coping as I can, but, when it comes to those kind of stuff, emotional stuff, … I don’t have anyone to talk to, because I am going straight home, just close the door, and sleep and say, “Hey, that day has passed. Just start a new day. These things happen.”’

In addition, most of the participants stressed that they need personal development to develop coping skills in terms of their physical and emotional challenges, including knowledge of their rights in the workplace and counselling services. They felt that such skills and reasonable accommodation measures would go a long way in improving their physical, emotional, spiritual and occupational wellness. For instance, reasonable accommodation makes allowances for a person’s personal circumstances resulting from their disability (as in the last quote above, the need to remain or move with their family) (RSA, DOL 2015; RSA, DSD 2015). Through reasonable accommodation (modifications or adjustments to a job to accommodate the needs of EWDs), the employer can reduce some of the stress factors in the work environment, enabling EWDs to cope better in the workplace (RSA, DOL 2014b).

Counselling would also serve as personal development for EWDs as the need arises. Various participants stressed the importance of counselling services – not only to help them cope with disability but also with all the changes and challenges experienced. Participant E specified the value of counselling:

‘Such a big organisation like this should have something. You know, they do at the clinic and so on, the AIDS clinic … but they should have supporters though. But it’s not so … specifically aimed at disability, but in general.’

Finally, it also became evident that EWDs need development to better understand their rights and what reasonable accommodation entails. Both the researcher and co-coder found that the participants initially stressed that they experience high levels of wellness in all dimensions, whilst as the interviews continued, it became clear that these initial responses were because of a halo effect based on their gratitude of being employed. This also became clear in what participants were willing to accept or endure in the workplace although, in ‘normal’ circumstances, these measures would be considered a breach of human rights as is clear in the following remark by Participant A:

‘Look, there’s a toilet … on the 27th floor …, [*a*] disabled toilet that I am taking a lift and going there. It is wide open, … I think everything is coming perfect.’

This employee showed that he is willing and even thankful to take a lift and ‘travel’ through various floors and quite a distance to an accessible restroom. However, he then also acknowledged that in the case of a power failure or load shedding, he would be left stranded with no access to such or in effect, any restroom.

#### Category 2: Education, training and development is also required for organisations

The participants referred to the need for line managers, wellness managers and all employees in an organisation to be trained on disability issues.

### Management development

A common need identified by most participants was the need for managers to receive disability training, not limited to sensitisation but also on reasonable accommodation. The participants stressed that managers tend to forget about the practical and psychological impacts of their decisions on EWDs. As mentioned before, participant G was relocated away from his family:

‘It happens that I fell down and then hurt my back …it happened during the Easter period. …the person who was in charge by that time, he questioned me whether it was true or not. …he went through, ‘Why didn’t you do this at this time?’ I mean, I was hurt. I felt that, because it seems like I was maybe telling [a] lie, even though I came with the doctor’s thing. [*Now*] I am scared to say, “Can I go and see a doctor? I am scared that it will be questioned.”’

Another participant with a physical impairment indicated that during the relocation of her organisation, public transport no longer dropped employees off at the offices. The participant was left waiting for any willing colleague to transport her the last stretch to the office:

‘… I just get off from the taxi and just stand at the corner, … everybody is just picking me up. One day I laugh at, aah, because when I was standing there about four cars of my colleagues … [laughing], my Manager just stood in front ….’

This showed that the participant’s manager was or became aware of the situation, but even at the time of the research interview the situation had not yet been addressed. Although the Toolkit for Employers in the Private Sector indicates that employers are not expected to provide transport for EWDs, unless it provides transport to all employees (South African Human Rights Commission 2015); in this case, the change and resulting challenge were because of a decision made by the employer.

Better trained managers would approach these situations differently and make informed decisions that do not impact negatively on the wellness of employees (Mellor & Webster 2013). For instance, in the last example, managers trained and educated in disability matters could have foreseen and addressed this challenge during the planning of the relocation or as soon as they became aware of it and realised the need for reasonable accommodation.

The *Technical Assistance Guide* on the *Employment* of PWDs (TAG) (RSA, DOL 2017) stresses that supervisors and managers also need training and education on the performance management of EWDs to ensure that they are assessed only on the key functions of their positions.

### Development of employees involved in health and wellness or diversity management

These employees are the first point of access for both EWDs and managers when they need assistance with disability issues. However, Participant E indicated that after the traumatic event that left him disabled, there was limited professional support available to him or his family:

‘Now, my question is, never, nobody ever went out and see if my wife was okay… I think the [*Executive*] phoned her once, but nobody came out and said, “Yes, are you alright?” And I always thought, “Why did … such a big organisation, why don’t they have a social welfare looking after people like that?”’

According to Marx (2015), employers also have a responsibility towards the families of employees in general and especially after suffering trauma. According to the latest public policy, employers should ensure that disability advocacy and expertise are available in organisations (RSA, DSD 2015). Therefore, these employees need to understand the disability management process and speak with authority on relevant public and organisational policy as well as reasonable accommodation.

Participant G captured this in the following response:

‘…companies on a whole, they have to now try and accommodate people with disabilities with regards to work-wise and stuff like that, and there’s got to be an improvement with that ….’

Whilst Participant L indicated that:

‘… they can maybe employ someone who … can respond our same position … same as us … disabled like us … can do those things for us.’

### Organisational development

The general lack of knowledge of disability and ‘disability etiquette’ by all employees was raised as an important developmental area to build relationships and interaction between EWDs and other employees. Participant A indicated that:

‘One thing remaining is the disability, but people should be taught how to treat people in a wheelchair. Let’s say, how to treat people on disability ….’

Whilst participant J indicated that other employees are:

‘… scared, and because they don’t know how to do it, they do it wrong, and then they feel bad, and they sort of just ignore the person or belittle him … I think people should be aware that a person with a disability does need some support … but not in a patronising way.’

The *White Paper on the Rights of Persons with Disabilities* (National Disability Policy) stresses that to address the discrimination against PWDs in the workplace, employers and all employees need disability sensitisation (RSA, DSD 2015). Organisational development also includes the mainstreaming of disability by ensuring that it is considered and included in all policies and procedures in an organisation (RSA, DOL 2015).

#### Category 3: Education, training and development for external stakeholders

**Society:** According to the WHO (2015, 2016), PWDs are still the most disadvantaged group in society. This not only refers to prejudice but also challenges like accessibility to basic services and even public transport, or in the case of their own transport, a lack of suitable parking facilities. According to the National Disability Policy prejudice is:

…the judgment or opinion that is formed without proper understanding or investigation, in a way that is biased, unfair, hurtful, and discriminatory. (RSA, DSD 2015:48)

Participant A indicated the following:

‘The municipality … Now what is their aim? What are they willing to help us? Look, you come to the [*Organisation*] here. Inside the building, there are disabled parkings … They can be able to access and move it easy to them … But then why is this not being done outside? I have seen two parkings in town as the whole CBD since I have drove … They cannot construct for taxis that loads people; they cannot construct for buses that carry people … When they think and do a bus for municipality that can be able to carry people with disability ….’

The South African government has acknowledged that there is still a great deal of discrimination against PWDs when considering accessible services, buildings and transport (RSA, DSD 2015). According to the Bill of Rights in the South African Constitution, nobody may be discriminated against based on their disability. Persons with disabilities therefore have a right to be treated equally and fairly and not to be excluded from any activity or even premises based on their disability (RSA, Department of Justice and Constitutional Development 1996). Therefore, their exclusion from accessible services, buildings and transport as mentioned above is considered discrimination.

To address this and to ensure more equal opportunities for PWDs, the government developed the National Disability Policy (RSA, DSD 2015).

Employers also have a responsibility to advocate the rights and needs of PWDs to not only show support for their EWDs but also to force government to address these needs. Furthermore, through sensitisation sessions in communities, they can help overcome the prejudices towards PWDs (RSA, DOL 2015; WHO 2016). Participant B observed that:

‘Yes, but they also used to organise some session for disabled people like me and organise … children or kids from … schools to come here and meet us. Then we … negotiate about … what is expected from disabled people, how to treat disabled people ….’

Unfortunately, employers can also hinder the promotion of transformation towards PWDs by showing a lack of acknowledgement of and clear support towards this previously disadvantaged group. To name but one example, an industry-leading auditing and consulting firm in South Africa proudly reports on its transformation journey, specifically stating that it is ‘… committed to empowering South Africa’s citizens and setting right the inequalities of the past through a sustainable transformation strategy that leaves no man – or woman – behind’ (2017). It only includes reference to progress made in terms of two previously disadvantaged groups, race and gender, with no reference to the third group, PWDs (2020). This is still quite common, and although this does not necessarily mean there is no support for PWDs or progress towards employing EWDs, it does not remind communities of or advocate towards advancing PWDs.

**Service providers:** Employers should also ensure that they use service providers in their company that respects the rights of PWDs and ensures their equal participation. To stress the importance hereof, employers can offer development opportunities to prospective service providers in terms of these rights and to sensitise them towards disability. Two participants stressed contradicting experiences. Participant I reported a positive experience:

‘Yes, I think the company did play its part by giving me the opportunity to … expand my knowledge then, yes, they did … I did attend a lot of training and a lot of courses. It is always accessible.’

Compared to an experience by participant D:

‘Yes, so, … they did organise a medical session … But, they made a mistake of not considering us as disabled people, because the truck was parked down there, which was very distant for us. Then there are also, up there, are staircases there, so … it was not accommodative that one.’

### Theme 2: A wellness framework for employees with disabilities must include the education, training and development needs unique to disability

Hettler developed the six-dimensional model of wellness in 1976 to acknowledge the influence of different factors on the wellness of a person (Hettler 1976). This approach, for that reason, formed an integral part of the conceptual framework for this study as the researcher aimed to consider the wellness of EWDs holistically.

The research study showed that EWDs, like everybody else, experience all six wellness dimensions, but with certain additional variables and, especially, stressors. As seen under Theme 1, this study was able to identify various common variables and needs influencing the wellness of EWDs and how ETD can positively influence their wellness. Theme 2 therefore naturally flowed from Theme 1 into the wellness framework for EWDs.

This framework focuses on which ETD interventions and support will make a positive contribution to the wellness of EWDs. As was established before, EWDs have different and unique needs and all relevant role players need to be trained and sensitised towards these needs. For EWDs to enjoy improved wellness in all six wellness dimensions, the discrimination and prejudice that are still prevalent towards PWDs, including EWDs, need to be addressed (RSA, DOL 2015; WHO 2016). This framework clearly identifies who needs what development to contribute to the wellness of EWDs. The identification of these ETD needs, and the role players involved stem from the data collected from research participants as analysed and discussed above. The relationships portrayed in this framework are in no way absolute as wellness is forever changing and its dimensions interdependent.

The framework, for instance, stresses that to improve the social wellness of EWDs, both the employees in question and the broader communities they function in, need development. Employees with disabilities and PWDs in general need to understand their role in their communities and what they can expect from other community members and the government to ensure their full participation in society. Communities should receive training and education on how to interact with PWDs and to help identify and address challenges that hinder this full participation (RSA, DOL 2015; RSA, DSD 2015).

**FIGURE 2:**
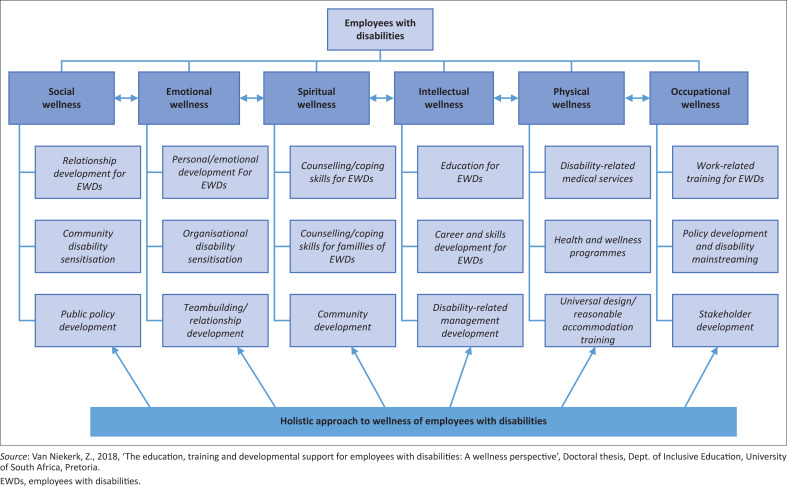
Wellness framework for employees with disabilities.

Figure 2 therefore displays the key concepts per dimension on which the EWDs or any other stakeholder, as specified, need ETD to address the wellness challenges experienced by EWDs. These wellness challenges can be found in their physical, emotional, social, occupational, intellectual and spiritual lives and, if not addressed, will lead to negative influences in the specific dimension of wellness and in the holistic wellness of a person. The unaddressed challenges will also impact on other people like their family, colleagues or community and spiritual stakeholders. The framework therefore attempts to best portray the findings in this study and, although it cannot be generalised, it can be a guide to a better understanding of the wellness of EWDs.

## Strengths and limitations

It is, however, of the utmost importance to note that people may be at different points in their wellness journey, and therefore, not all EWDs will need all training identified and employers must therefore use this as a guideline to consider every employee’s individual needs and circumstances.

## Conclusion

The study led to the following conclusions in terms of the research aim, question and themes discussed in this article:

- In line with the research aim for this study, EWDs shared their own wellness experiences in the workplace especially thankful to be employed but acknowledging the need to be developed in all wellness dimensions.
- Furthermore, the study also identified various other areas where managers, colleagues, society and other stakeholders need capacity ETD to positively contribute to the holistic wellness of EWDs.

This study considered wellness for EWDs. Two themes identified through content analysis after qualitative research interviews led to a proposed framework that can assist employers in developing all role players, including EWDs, to contribute to their physical, emotional, social, occupational, intellectual and spiritual dimensions of wellness. As these interrelated dimensions improve, so will the holistic wellness of EWDs.

## Recommendations

Based on the above discussion and findings, the following recommendations are made:

- Employers should actively ensure that EWDs are exposed to ETD opportunities that will address the discrimination and lack of development the PWDs have been exposed to. Employers should also offer opportunities to all role players to contribute to all wellness dimensions experienced by EWDs.
- The ETD interventions offered by employers should help both EWDs and managers understand disability legislation, rights and concepts like a reasonable accommodation.
- Employers should offer development opportunities within and outside the workplace to create disability awareness and to promote the rights of EWDs, including their relationships with service providers and the wider communities.
- All role players, including employers, community leaders, government and PWDs, need to actively pursue the promotion and enforcement of all new disability-related public policies and legislation declared since 2014.
- Public policy measures and disability in general should be included in organisational policies and operational plans, whilst the organisation should actively pursue equality for EWDs inside and all PWDs outside the organisation.
- More research should be done on the wellness of EWDs and input from employers should also be considered.
- Employees with disabilities must become their own biggest champions by identifying and knowing their own needs, rights and – through increased participation – other challenges.
- On a more practical level, employers should take all reasonable steps to address the challenges EWDs experience in their workspace. Employers will need to ensure that facilities like accessible restrooms are readily available to EWDs or make formal arrangements to ensure that EWDs reach their office safely, especially where alternatives like public transport are not available. Employers need to develop an all-encompassing disability policy that includes the process for EWDs to apply for reasonable accommodation. It would be suitable for such a policy to provide sourcing expert inputs in considering these applications.
